# Enhanced Photocatalytic Coupling of Benzylamine to N-Benzylidene Benzylamine over the Organic–Inorganic Composites F70-TiO_2_ Based on Fullerenes Derivatives and TiO_2_

**DOI:** 10.3390/molecules28114301

**Published:** 2023-05-24

**Authors:** Yanmeng Guo, Hang Li, Bo Li, Shizhuo Su, Xin Zhong, Derui Kong, Yifan Chen, Yujie Song

**Affiliations:** Hainan Provincial Key Laboratory of Fine Chemicals, College of Chemical Engineering and Technology, Hainan University, Haikou 570228, China; gym15799033080@163.com (Y.G.); 20203100907@hainanu.edu.cn (H.L.); l1178938050@163.com (B.L.); 20190411310066@hainanu.edu.cn (S.S.); zhongxin20210831@163.com (X.Z.); 20085600210093@hainanu.edu.cn (D.K.)

**Keywords:** organic–inorganic composites, photocatalytic coupling, fullerene derivatives, TiO_2_, sol–gel method

## Abstract

The organic–inorganic composites F70-TiO_2_, based on fullerene with carboxyl group derivatives and TiO_2_ semiconductor, have been designed and constructed to become an optical-functional photocatalyst via the facile sol–gel method. The composite photocatalyst obtained shows excellent photocatalytic activity for the high-efficiency conversion of benzylamine (BA) to N-benzylidene benzylamine (NBBA) with air pressure at a normal temperature under visible light irradiation. By optimizing the composition, the composites with the 1:15 mass ratio of F70 and TiO_2_, denoted as F70-TiO_2_(1:15), demonstrated the highest reaction efficiency for benzylamine (>98% conversion) to N-benzylidene benzylamine (>93% selectivity) in this study. However, pure TiO_2_ and fullerene derivatives (F70) exhibit decreased conversion (56.3% and 89.7%, respectively) and selectivity (83.8% and 86.0%, respectively). The UV–vis diffuse reflectance spectra (DRS) and Mott–Schottky experiment’s results indicate that the introduction of fullerene derivatives into anatase TiO_2_ would greatly broaden the visible light response range and adjust the energy band positions of the composites, enhancing the sunlight utilization and promoting the photogenerated charge (e^−^-h^+^) separation and transfer. Specifically, a series of results on the in situ EPR tests and the photo-electrophysical experiment indicate that the separated charges from the hybrid could effectively activate benzylamine and O_2_ to accelerate the formation of active intermediates, and then couple with free BA molecules to form the desired production of N-BBA. The effective combination, on a molecular scale, between fullerene and titanium dioxide has provided a profound understanding of the photocatalysis mechanism. This work elaborates and makes clear the relationship between the structure and the performance of functional photocatalysts.

## 1. Introduction

Imines are critical building blocks in the field of fine chemicals and pharmaceuticals [1,2]. Imines are typically prepared by the condensation reaction between amines and active carbonyl derivatives with the assistance of Lewis acids or bases, which causes plenty of undesirable chemical wastes and increases the difficulty of post-processing, resulting in environmental contamination [3,4]. Consequently, it is necessary to research green alternatives for synthetic routes and sustainable processes to meet the rising energy demand and the many strict environmental policies. In recent years, the heterogeneous photocatalysis, based on functional semiconductor materials, has garnered remarkable attention as a green approach for imines fine chemicals synthesis, such as with oxygen or air as the oxidant [5,6], which is an energy-saving, environmentally friendly and easily recyclable process. Among them, many researchers have focused on TiO_2_^−^-based photocatalysts due to their low-cost, non-toxicity, high stability and excellent photocatalytic activity [7]. However, the wide band gap (~3.2 eV, which corresponds to about 4% of solar energy received) of TiO_2_ means that TiO_2_ could not utilize the visible light (about 50% of the received solar energy) to initiate the photocatalytic reaction, greatly limiting the utilization of solar energy. Moreover, photogenerated charges occurring in TiO_2_ are extremely easy to recombine, leading to photocatalytic activity abatement, which also hinders the application of TiO_2_ in the photocatalytic field [8,9,10]. To tackle this issue, many efforts have been dedicated to exploiting the visible-light-active TiO_2_^−^-based photocatalysts with outstanding photogenerated charge pairs separation, such as metal deposition (Pt [11], Pd [12], Au [13], Ni [14]), doping non-metal atoms (C, N, S) [15,16] and photosensitization [17,18,19]. However, the promotion of photocatalytic activity on the catalysts modified by the above strategy is limited, which is attributed to the narrow spectral response range or poor photocatalytic stability. Therefore, it is necessary to explore a facile and practical combination strategy for constructing TiO_2_-based composite materials with excellent optical-response ability and robust stability.

Conjugated materials are potential organic semiconductor candidates for widening the optical response range and facilitating photogenerated charges separation and transfer during the photocatalytic process by the formation of electronic–chemical interactions with TiO_2_ [20,21]. Among them, fullerenes (C60) derivatives have garnered considerable attention for their excellent optical-electronic, physical and chemical properties owing to their unique three-dimensional cage with huge conjugated delocalized structures and their strong electron-accepting ability [22]. Furthermore, the C60-based functional materials, with excellent exciton mobility and a rather long exciton diffusion distance, would effectively stimulate photoexcited charge separation and greatly suppress charge recombination [23,24,25,26]. Thus, the effective combination of C60 derivatives with semiconductor materials has been explored to boost the performance in photovoltaic conversion applications [27]. In particular, the C60-based organic–inorganic composites are mainly applied in the field of photocatalytic organic pollutant degradation. The related mechanism research shows that the unique huge π-conjugated structure of fullerene can efficiently accumulate electrons and regulate the energy band position of semiconductor materials, resulting in the rapid separation of photogenerated charges (e^–^−h^+^) to decline the charge recombination. Furthermore, the excited electrons from the charge separation could be enriched and then stimulate the photoreduction of O_2_ for •O_2_^−^ active species. The •O_2_^−^ radical production could activate many organic reactions [7,28]. Therefore, the composites based on C60 and the metal oxides semiconductor have potential applications in the organic photooxidation reaction. However, there is little related research work, particularly, on the photocatalytic coupling reaction of amines. The main reason is that no effective synthesis strategy has been put forward to obtain stable and high-activity C60-based composite materials. At present, research results display that the combination strategies of the composites based on C60 and TiO_2_ are impregnation and sensitization, resulting in C60 only absorbing onto the surface of TiO_2_, which not only confines the loading capacity of C60 but also reduces the stability of composites owing to the fact that C60 extremely easily degrades and drops off the surface of TiO_2_. Therefore, it is significant to take an appropriate approach to promote the stability of composites and regulate the component ratio as much as possible for modifying the photocatalytic oxidation coupling activity of amines.

Our earlier findings reveal that the organic–inorganic hybrids based on TiO_2_, and organic optical functional molecule obtained by the in-situ sol–gel method, exhibit remarkable stability and a gratifying synergistic photocatalytic activity in terms of broadening the spectral response range and promoting photogenerated charge separation and transfer [29,30,31,32]. the organic dopants could rely on their active binding sites (-COOH, -OH, -NH_2_ and -PO_3_H_2_, etc.) by in-situ sol-gel method to improve the uniformity and compatibility of organic–inorganic hybrid materials, [33,34], which could undergo dehydration and a condensation reaction with titanium precursors, such as tetra butyl titanate and titanium tetrachloride, to form covalent graft composites. Compared with the conventional sensitization system, the organic and inorganic components in the hybrid crosslink with each other to form a network structure, which effectively fixes and confines the organic substrate in space, to achieve excellent photocatalytic activity and durable photocatalytic recycling stability for the hybrid [35,36,37].

In this work, a series of C60 derivatives were modified by TiO_2_ via a facile sol–gel method. C60 derivative with -COOH active sites (denoted as F70) and TiO_2_ were robustly combined through the covalent chemical bonds to give an organic–inorganic hybrid, denoted as F70-TiO_2_. Furthermore, the hybrid showed a dramatic activity and stability for the photooxidizing coupling of amines to imines using air as oxidant, which is attributed to the introduction of F70, considering the following advantages: (1) the introduction of electron-accepting units (-COOH) in F70 enhances the capture of electrons and ensures a more effective electron separation and transfer between fullerenes and TiO_2_; (2) the presence of -COOH groups in F70 endows the hybrid with outstanding light responsiveness and a low conduction band position, which further facilitates the separation of the photogenerated charge with visible-light excitation; (3) the -COOH active groups of F70 could interact with Ti(OC_4_H_9_)_4_ to form the robust titanium ester bond Ti-O-C=O-C60 bonds, which preserves the stability of the hybrid system in the process of the photocatalytic reaction; (4) in the hybrid, F70 acts as an electron reservoir, which could provide a continuous charge supply for the production of •O_2_^−^. The related structural characteristics and photocatalytic mechanism on fullerenes derivative-modified TiO_2_ were verified by a series of characterizations.

## 2. Results and Discussion

**Figure 1:**
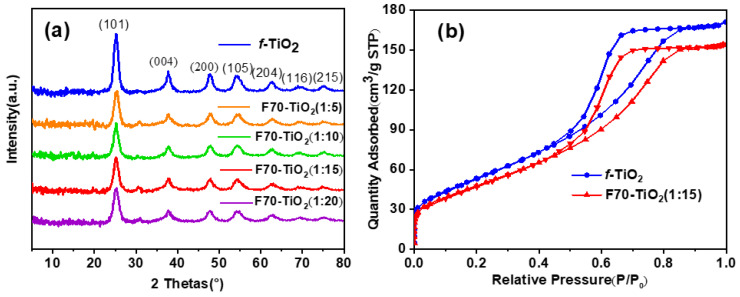
(**a**) The XRD patterns of F70-TiO_2_ with different F70 content were recorded, (**b**) N_2_ adsorption-desorption isotherms of *f*-TiO_2_ and F70-TiO_2_(1:15).

The crystallographic features of synthesized F70-TiO_2_ composites and *f*-TiO_2_ were investigated by XRD and are displayed in Figure 1a. The relevant XRD signal peak at the top corresponds to anatase TiO_2_ [29]. In addition, no obvious XRD signal peaks of F70 in the composites were found. Thus, the introduction of F70 hardly had an impact on the crystallinity of TiO_2_, owing to the low doping contents of F70. The BET surface areas and porosity distribution of photocatalysts in this work were assessed by a N_2_ adsorption measurement at 77 K. As shown in Figure 1b, the adsorption isotherms of *f*-TiO_2_ and F70-TiO_2_(1:15) were ascribed to a typical type IV pattern with surface areas of 198.6 m^2^ g^−1^ and 178.8 m^2^ g^−1^, respectively. The pore sizes distributions of *f*-TiO_2_ and F70-TiO_2_(1:15) were calculated to be about 4.39 nm and 4.45 nm based on the Barrett–Joyner–Halenda (BJH) (Table S1), which agrees well with the BET surface areas of *f*-TiO_2_ and F70-TiO_2_(1:15).

**Figure 2:**
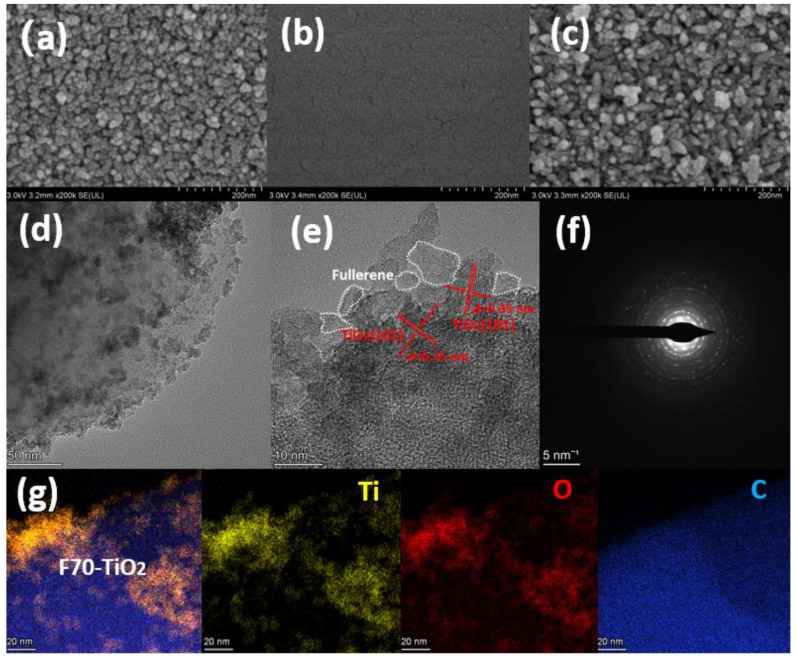
(**a**) HRSEM of *f*-TiO_2_, (**b**) HRSEM of F70, (**c**) HRSEM, (**d**) TEM, (**e**) HRTEM, (**f**) SAED and (**g**) the elemental mappings image of F70-TiO_2_(1:15).

Furthermore, SEM images revealed the distinction of samples on the morphology. Among them, F70 displayed an amorphous planar microstructure (Figure 2b and Figure S3b). Additionally, *f*-TiO_2_ showed an agglomeration of spherical nanoparticles with a size of 10–20 nm (Figure 2a and Figure S3a), whereas SEM images of F70-TiO_2_(1:15) (Figure 2c and Figure S3c) showed that the composites mainly consist of ellipsoidal nanoparticles and fine irregular nanoparticles, demonstrating that the introduction of F70 into TiO_2_ could uniformly modify the nanoparticle’s shape, size and the porosity morphology of TiO_2_, which could be confirmed by the TEM images and the elemental mapping images (Figure 2d,e,g and Figure S3d,f). The distances observed, of 0.35 nm, at two adjacent lattice fringes were attributed to the (101) plane of TiO_2_ (Figure 2e). The SAED pattern of F70-TiO_2_(1:15) nanomaterials proved again the existence of the TiO_2_ anatase phase, corresponding to the XRD result (Figure 2f). The EDX analysis further demonstrated the existence of F70 in the hybrids (Figure S4), which corresponds with the XPS results. In Figure S5, the XPS spectra of F70-TiO_2_(1:15) displayed Ti2p_1/2_ and Ti2p_3/2_ peaks situated at 458.7 eV and 464.5 eV, attributed to Ti^4+^, which are slightly redshifted with respect to pure TiO_2_ [38]. The doping of fullerene into TiO_2_ caused electrons around the Ti-O bond flowing away from the Ti atom so that the binding energy of Ti2p slightly increased, due to the strong electron-absorbing capability of fullerene. Compared to the compositions of 24.5% Ti, 51.8% O and 23.7% C for *f*-TiO_2_, the compositions of 21.7% Ti, 44.9% O and 33.4% C for F70-TiO_2_(1:15) with the excess C percentage were obtained to confirm the compositional distinction of the hybrid and the pure TiO_2_, which was derived from the introduction of F70 into the hybrid (Table S2).

**Figure 3:**
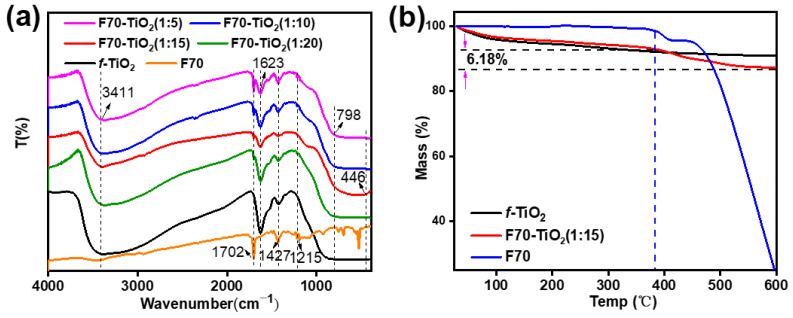
(**a**) FT-IR spectra of *f*-TiO_2_ and series of F70-TiO_2_ hybrid samples. (**b**) Thermal gravity analysis of *f*-TiO_2_, F70, and F70-TiO_2_(1:15) hybrid materials.

Moreover, the FT-IR spectrum of F70-TiO_2_ showed that the hybrid is composed of F70 and *f*-TiO_2_. In Figure 3a, the typical stretching vibrations peaks of C-O and C=O groups on F70 are at 1080–1208 cm^−1^ and 1702 cm^−1^, respectively, which appears along with the mass increase in F70 in the composites. The characteristic vibration bands of -OH groups on the surface of TiO_2_ are around 1623 cm^−1^ and 3411 cm^−1^, and the vibration bands of Ti-O from TiO_2_ are at 446–798 cm^−1^, existing in a series of composites, which indicates that TiO_2_ could combine with F70 through the condensation reaction between hydroxyl group and carboxyl group. However, the above characterizations are qualitative or semi-quantitative to confirm the relationship between F70 and *f*-TiO_2_. For the quantitative analysis of the composite ratio of F70-TiO_2_(1:15) composite, a thermogravimetric analysis of samples was conducted at an air atmosphere from 25 °C to 600 °C with a heating rate of 10 °C/min. The thermogravimetric analysis plots of F70, *f*-TiO_2,_ and F70-TiO_2_(1:15) are shown in Figure 3b. This indicates that F70 begins to decompose at about 383 °C. However, the quality of TiO_2_ did decrease significantly up to 600 °C. For the hybrid F70-TiO_2_(1:15) sample, the analyzed decomposition temperature is about 383 °C to 550 °C, corresponding to the mass loss of 6.18% for the hybrid, which is consistent with the mass ratio of F70 in the F70-TiO_2_(1:15) sample (with about 6.28% mass ratio of F70 in the hybrid in theory). Therefore, this proves that F70 is combined successfully with TiO_2_ by the sol–gel method and the hybrid has stable structure and components.

**Figure 4:**
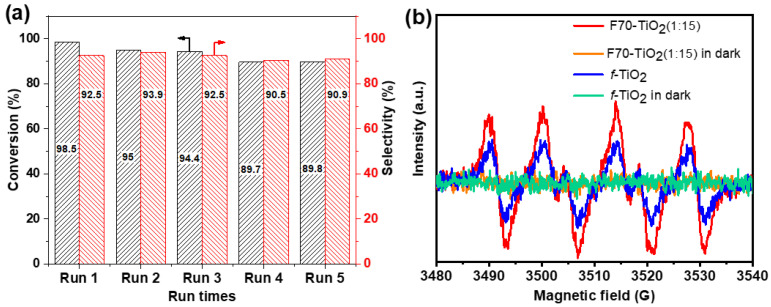
(**a**) Conversion and selectivity of benzamine oxidation over the F70-TiO_2_(1:15) photocatalyst. (**b**) ESR detection of •O_2_^−^ generated by *f*-TiO_2_ and F70-TiO_2_(1:15) with DMPO as the •O_2_^−^ trapping agent. Reaction conditions: BA (0.2 mmol), catalyst (5 mg), CH_3_CN (1.5 mL), Air (1 atm), λ > 400 nm, 12 h.

A series of experiments based on the mathematical theory model were carried out to explore the photocatalytic performances on the photooxidative coupling of BA to N-BBA at atmospheric pressure with visible light irradiation. The results are listed in Table 1. Under the same conditions, the hybrid with the different ratios of F70 and TiO_2_ shows excellent photocatalytic performance. As the mass ratio of F70 and TiO_2_ in the hybrid increases from 1:20, 1:15 to 1:10, the BA conversion performance successively increases; F70-TiO_2_(1:15) exhibits the highest conversion up to 98.5% with the highest selectivity for N-BBA (93.9%) (Table 1, entry 4) with the GC-MS spectrogram confirming the relative molecular mass for production (in Figure S6). However, with further increase in the mass ratio of F70 and TiO_2_ in the hybrid to 1:10 and 1:5, the photocatalytic activity of F70-TiO_2_ successively decreases, as shown in the table. In comparison, *f*-TiO_2_ shows a low conversion rate of BA (56.3%) and selectivity rate of N-BBA (89.7%) (Table 1, entry 1), and F70 has 83.8% conversion for BA with 86.0% selectivity for N-BBA (Table 1, entry 7). Furthermore, light is a very necessary condition. F70-TiO_2_ only has a conversion rate of 18.4% and a selectivity of 6.36% in the absence of light (Table 1, entry 5). These results suggest that fullerene structure with a significant electron enrichment ability could advance photogenerated charge separation, which further promote the conversion of BA. In addition, previous research suggests that the introduction of Pd and Pt into the catalyst system could further facilitate the formation of N-BBA from BA [39]. However, for F70-TiO_2_(1:15) composites loaded with a mass ratio of 1.0%Pd, no effective promotion in the photocatalytic activity was traced, compared to F70-TiO_2_(1:15) without Pd nanoparticles loaded. On the contrary, the selectivity of N-BBA (88.2%) (Table 1, entry 8) was reduced, which reveals that fullerene plays an important role in the photocatalysis process. Therefore, F70 is a photocatalytic functional regulator in the hybrid, which not only works as an organic photosensitizer to broaden the spectral response range but also acts as an organic semiconductor or electron accumulator to facilitate photogenerated charge separation. Furthermore, the recycle stability of F70-TiO_2_(1:15) was determined via five recycles on the photocatalytic oxidation coupling reaction from BA to N-BBA under the same conditions as described above. As shown in Figure 4a, F70-TiO_2_(1:15) maintained a high photocatalytic performance in the transformation of BA to the desired molecule N-BBA during the five cycles process, which confirms the structural and performance stability of the prepared photocatalyst. This is verified by the XRD signal peaks of F70-TiO_2_(1:15) recycled after multiple cycles test in Figure S7, which retains similar signal peaks shifts with the hybrid materials before the recycle test.

Previous studies show that O_2_ molecules are necessary in the photooxidative coupling route from BA to N-BBA. To fully explore the influence of the active species and the intermediate states of the O_2_ molecule, EPR measurements were implemented [40], as shown in Figure 4b. No obvious EPR signals were monitored for *f*-TiO_2_ and F70-TiO_2_(1:15) under darkness. However, under visible light irradiation, intense EPR signals were detected for F70-TiO_2_(1:15) with DMPO as the trapping agent [41], owing to the existence of •O_2_^−^ species. Compared with F70-TiO_2_(1:15), *f*-TiO_2_ showed weak EPR signal intensity under visible light irradiation, indicating that more •O_2_^−^ species are generated over F70-TiO_2_(1:15). These results reveal that the introduction of fullerene could facilitate the photogenerated e^–^separation from charge pairs and then transfer on the interface of F70 and TiO2 to enhance the photoreduction of O_2_ to •O_2_^−^ active species. In addition, p-benzoquinone (BQ) and AgNO_3_ could work as scavengers to trap and neutralize •O_2_^−^ and e^–^, respectively. After the addition of 1 mM AgNO_3_ or BQ, the yield of N-BBA for the catalyst F70-TiO_2_(1:15) decreases significantly to only 11.6% and 14.8%, respectively. Furthermore, without light irradiation, F70-TiO_2_(1:15) only has 11.0% of the conversion with 6.34% selectivity for N-BBA, which demonstrates that a light source is essential for the photooxidative coupling from BA to N-BBA. Therefore, light source, photogenerated e^–^ and •O_2_^−^ play major roles in the photocatalytic coupling reaction process

**Figure 5:**
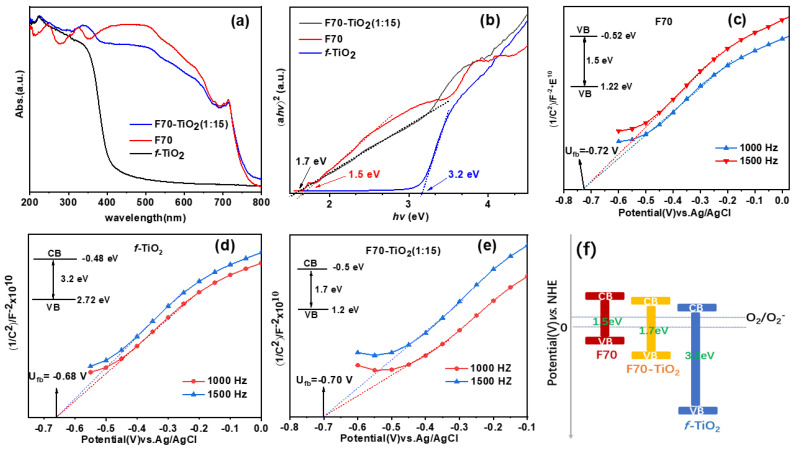
(**a**) The UV–vis DSR and (**b**) the Tauc plot of *f*-TiO_2_, F70, and F70-TiO_2_(1:15). (**c**) The Mott–Schottky plots of F70. (**d**) The Mott–Schottky plots of *f*-TiO_2_. (**e**) The Mott–Schottky plots of F70-TiO_2_(1:15). (**f**) The energy band position schematic of *f*-TiO_2_, F70 and F70-TiO_2_(1:15).

**Figure 6:**
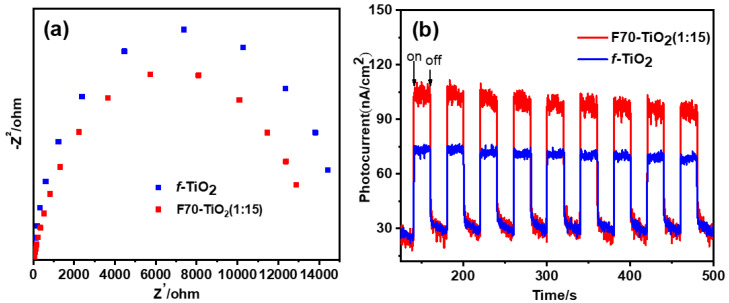
(**a**) The EIS spectra and (**b**) the photocurrent intensity of *f*-TiO_2_ and F70-TiO_2_(1:15).

A series of photo-electrophysical and photo-electrochemical measurements were executed to explore the root of the improved photocatalytic activity on F70-TiO_2_ composites. In Figure 5a, the UV–vis DRS shows that *f*-TiO_2_ has a relatively narrow optical absorbance range below 400 nm, while the light absorbance range of F70 continued extending to 750 nm. It is worth noting that F70-TiO_2_ composites also exhibit a similar broad absorbance range at 750 nm, and a slightly weaker intensity than F70, which inherits the optical trait of F70 and TiO_2_. This suggests that the doping of F70 in the hybrid would positively regulate the optical absorbance responsiveness for the efficient utilization of sunlight. To further reveal the possible mechanism of the photocatalytic reaction routes, the M-S plots of *f*-TiO_2_, F70 and F70-TiO_2_ were detected to obtain the flat-band potential. As in previous works, it is generally believed that the conduction band (CB) potential of the n-type semiconductor was almost equal to the flat-band potential [29]. In Figure 5b–d, the flat-band potentials of *f*-TiO_2_, F70 and F70-TiO_2_ were ascertained to be about −0.68 V, −0.72 V and −0.70 V vs. Ag/AgCl, respectively. Thus, the CB of *f*-TiO_2_, F70 and F70-TiO_2_ were calculated as −0.48 V, −0.52 V and −0.50 V vs. NHE, respectively. All of them were more negative than the redox potential of O_2_/•O_2_^−^ (E_re_ = −0.04 vs. NHE) [42], which is thermodynamically feasible for oxygen reduction. In addition, the band gap values of *f*-TiO_2_, F70 and F70-TiO_2_ were estimated about 3.2 eV, 1.5 eV and 1.7 eV, respectively, based on the results of the UV–vis DRS, as shown in Figure 5e. Therefore, the valence band (VB) potential could be calculated as 2.7 eV, 0.98 eV and 1.2 eV vs. NHE, which is thermodynamically feasible for the benzylamine oxidation (E_ox_ = +0.76 vs. NHE) [42]. Moreover, the energy band structure and position of *f*-TiO_2_, F70, and F70-TiO_2_ were depicted, as shown in Figure 5f. This indicates that the F70-TiO_2_ composite is a classic heterojunction semiconductor, and that F70 would greatly regulate and change the energy band structure and position of the hybrid, which is attributed to the unique electron-rich conjugated structures of F70 as a functional regulatory semiconductor. The physical energy band theory shows that the charge transfer between interfaces plays an important role in decreasing the photogenerated charges recombination. Furthermore, EIS was performed to evaluate the internal resistance of the catalysts at the interface during the charge separation process (Figure 6a). Compared with *f*-TiO_2_, F70-TiO_2_ has a smaller arc radius in EIS plots, indicating a lower charge transfer resistance at the interface, which is ascribed to the robust linkage that exists between F70 and *f*-TiO_2_. Similarly, F70-TiO_2_ has a higher photocurrent density than the *f*-TiO_2_ sample (Figure 6b), indicating an effective photogenerated charge formation and transfer on the heterojunction composite. Given the almost unchanged BET surface area of F70-TiO_2_ and *f*-TiO_2_, the surface area for the F70-TiO_2_ composite is not the main factor affecting the photocatalytic activity. According to the aforementioned characterization results, the enhanced photocatalytic activity of the F70-TiO_2_ composite may originate from the widened light absorption range and the efficient charge separation and transfer in the hybrid.

**Scheme 1:**
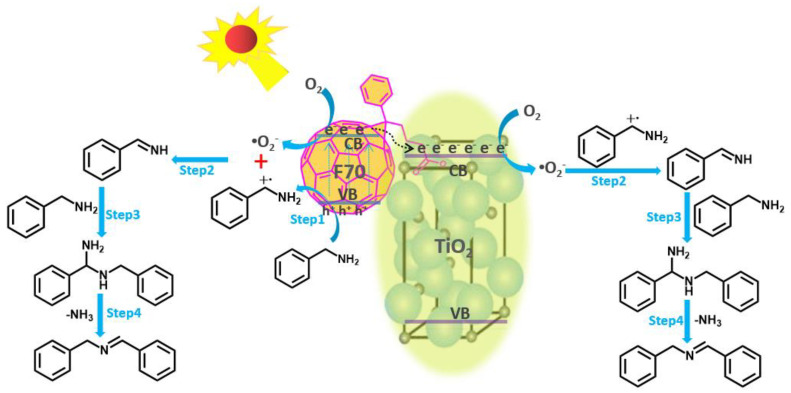
Proposed mechanism of photocatalytic coupling oxidation of F70-TiO_2_ over visible-light irradiation.

Based on the results above, a possible photocatalytic mechanism on imine formation under aerobic conditions is illustrated in Scheme 1. Under visible-light irradiation, the photogenerated electrons on the hybrid are excited and jump to the conduction band of F70, leaving holes on the value band of F70. The band structure and the energy potential positions demonstrate that F70 possesses enough oxidative and reductive potential to oxidize the benzylamine into a benzylamine cationic radical formation (E_OX_ = +0.76 vs. NHE) and reduce oxygen molecular to active species (E_re_ = −0.04 vs. NHE). Thus, the excited electrons transfer onto the surface of TiO_2_ to reduce O_2_ to •O_2_^−^, which could realize electron–hole pairs’ separation and migration efficiently on the composite, while benzylamine is oxidized by the valence band hole to obtain benzylamine radical cation (step 1). Then, the benzylamine cationic radical reacts with the active •O_2_^−^ radicals and transforms, subsequently, to Ph-CH=NH intermediate (step 2). Furthermore, the Ph-CH=NH intermediate could fleetingly react with a benzylamine molecule to form N-benzyl-1-phenylmethanediamine intermediate (step 3). Finally, the ammonia group of the intermediate is eliminated by holes, and the target product N, N-dimethylbenzylamine (N-BBA), is obtained (step 4).

## 3. Experiments

### 3.1. Materials

The relevant reagents utilized in this paper were purchased and used directly without further treatment. Triethanolamine (TEOA, >99.0%), terabutyl titanate (TBTA ≥ 99.0%), N, N-dimethylformamide (DMF, 99.9%), acetic acid (≥99.5%), acetonitrile (99.9%), silver nitrate (AgNO_3_) and 1,4-Benzoquinone, benzylamine were bought from Innochem company (Beijing, China). [6,6]-Phenyl C61 butyric acid (F70, 98%) was purchased from Beijing Allmers chemical S&T company (Beijing, China).

### 3.2. Synthesis of F70-TiO_2_ Materials

The structure of F70 and the preparation process of F70-TiO_2_ are shown in Figure S1. To prepare F70-TiO_2_, DMF (2.0 mL), distilled water (0.20 mL), acetic acid (0.30 mL) and 2.0 mL tetrabutyl titanate were subsequently put into a sample bottle to form a transparent sol substance, and then F70 with different mass contents (0/11.7/15.6/23.4/46.8 mg) was added and stirred. The mixture changed from light yellow transparent sol to black gel. After heating at 65℃ overnight, a black solid powder was obtained. Then, the residue was washed with distilled water for 72 h. Finally, the samples were evaporated to complete dryness to obtain a black powder. The series of catalysts, obtained using the above method with different F70 mass contents (0/11.7/15.6/23.4/46.8 mg), were denoted as *f*-TiO_2_, F70-TiO_2_(1:20), F70-TiO_2_(1:15), F70-TiO_2_(1:10) and F70-TiO_2_(1:5), respectively.

### 3.3. Photocatalytic Studies

The photocatalytic oxidation coupling reaction of benzylamine (BA) was conducted in a 10 mL Schlenk tube in an air atmosphere with visible light irradiation. The mixture of 0.2 mmol BA and 5 mg photocatalyst samples were added into a 1.5 mL acetonitrile solvent. Next, the mixture was sonicated for 15 min to mix evenly. Then, the mix was irradiated by a 300 W Xe lamp with a cutoff filter to obtain visible light (λ > 400 nm). The optical power density (~1.1 W cm^−2^) of the Xe lamp was measured by the light intensity meter. After 12 h irradiation, the mixture was filtered with a nylon needle filter. The supernatant solution was analyzed by a Shimadzu Gas Chromatograph (GC-2014C) with an RTX-5 capillary column. The results are shown in Table 1 and Figure S4. The conversion rate of BA and the selectivity rate on N-benzylidene benzylamine (N-BBA) are defined below:

$$Conversion\left(\%\right)=\left[\left(C_{0}-C_{1\;}\right)/C_{0}\right]\times 100\%$$

$$Selectivity\left(\%\right)=\left[C_{2}\times 2/\left(C_{0}-C_{1}\right)\right]\times 100\%$$

where *C*_0_, *C*_1_ and *C*_2_ are the initial concentration of BA, and the concentration of BA and N-BBA were determined by chromatography, respectively.

### 3.4. Materials Characterization

UV–vis absorption spectra of solid powder were tested by a Shimadzu UV-2600 spectrometer (Shimadzu, Kyoto, Japan). The Fourier Transform Infrared (FT-IR) Spectrogram was recorded on a Nicolet iS50 spectrometer (Thermo Fisher Scientific, Waltham, MA, USA). Powder X-ray diffraction (PXRD) studies were performed on a Rigaku Smart Lab with a diffractometer (Bragg-Brentano geometry, Cu-KA1 radiation, λ = 1.54056 Å) (Rigaku, Beijing, China). Scanning electron microscope (SEM) and high-resolution field emission scanning electron microscope (HRSEM) were used to record the morphology on a SU8020 (HITACHI, Tokyo, Japan). Transmission electron microscopy (TEM) images, high-resolution transmission electron microscopy (HRTEM) images, energy dispersive X-ray spectroscopy (EDX), high-angle annular selected area electron diffraction (SAED) patterns and elemental mapping images were achieved by an FEI Talos F200X transmission electron microscope (FEI, Hillsboro, OR, USA). X-ray photoelectron spectroscopy (XPS) measurements were performed using a monochromatic Al Kα X-ray radiation source with 1.487 keV (Thermo escalab 250Xi, Thermo Fisher Scientific, Waltham, MA, USA). Gas adsorption tests were carried out on the Auto-sorbiQ2-MP analyzer (Quantachrome, Beijing, China) with ultra-high purity N_2_. Thermogravimetric (TG) analysis was performed on a thermal analyzer (449 F5/F3 Jupiter NETZSCH, Free State of Bavaria, Germany) from 30 to 600 °C in air. The Mott–Schottky curves, the electrochemical impedance measurements and the photocurrent response (300 W xenon lamp with 400 nm cut-off filter as the light source) were performed on the electrochemical analyzer (Ivium Technologies B.V., Eindhoven, The Netherlands) with a three-electrode cell. To prepare the working electrolyte, samples were added to the solution of Nafion in ethanol. The electrolyte was 0.25 M Na_2_SO_4_ solution. Ag/AgCl electron with saturated KCl electrolyte solution was used as a reference electrode, and the platinum plate worked as a counter electrode.

## 4. Conclusions

In summer, a series of organic–inorganic hybrid materials based on fullerene derivatives and TiO_2_ were prepared by the facile sol–gel method. Among them, catalyst F70-TiO_2_ (1:15) (the mass ratio of F70 and TiO_2_) had an outstanding photocatalytic transformation and high selectivity for the oxidative coupling of the benzylamine (BA) to N-benzylidene benzyl-amine (N-BBA) at atmospheric pressure under visible light irradiation. The results on characterization of morphology, structure and mechanism shows that an excellent synergistic effect exists between the component of the hybrid in comparison to the individual components. The broad light adsorption ability and excellent photogenerated charge separation ability for the hybrid F70-TiO_2_ (1:15) were the key factors to obtain sufficient reactive species (h^+^ and •O_2_^−^) in the photooxidation process in this work. Differing from the usual photocatalytic reaction with noble metals, the introduction of fullerene, as a functional carbon material, provides a new perspective for the development of nitrogen-containing fine chemical products. With the constant consumption of noble metals, attention is gradually being paid to projects on artificial carbon-containing functional materials in order to explore their development and application in the photocatalytic field.

## Figures and Tables

**Table 1:** Photocatalytic oxidation of BA to N-BBA under various conditions.

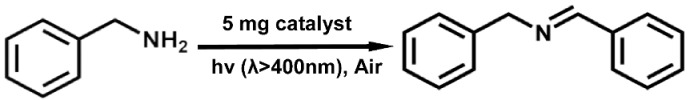				
**Entry**	**Catalysts**	***hv***	**Conv. (%)**	**Select. (%)**
1	*f*-TiO_2_	*+*	56.3	89.7
2	F70-TiO_2_(1:5)	+	94.4	77.1
3	F70-TiO_2_(1:10)	+	98.1	88.1
4	F70-TiO_2_(1:15)	+	98.5	93.9
5	F70-TiO_2_(1:15)	−	18.4	6.36
6	F70-TiO_2_(1:20)	+	97.6	83.1
7	F70	+	83.8	86.0
8	1%Pd/F70-TiO_2_(1:15)	+	96.6	88.2

Reaction conditions: BA (0.2 mmol), catalyst (5 mg), CH3CN (1.5 mL), Air (1 atm), λ > 400 nm, 12 h.

## Data Availability

The data presented in this article are available on request from the corresponding author.

## Supplementary Materials

The following supporting information can be downloaded at: https://www.mdpi.com/article/10.3390/molecules28114301/s1, Figure S1: the structure of F70 and Synthesis of F70-TiO_2_(1:15); Figure S2: The XRD signal peaks of F70; Figure S3: (a) SEM of f-TiO_2_, (b) SEM of F70, (c) SEM, (d) TEM, and (e) HRTEM of F70-TiO_2_(1:15); Table S1: the BET surface area and the pore size distributions of *f*-TiO_2_ and F70-TiO_2_(1:15); Figure S4: the EDX of F70-TiO_2_(1:15); Table S2: The Atom percentages and the binding energy of *f*-TiO_2_ and F70-TiO_2_(1:15) by the XPS analysis; Figure S5: XPS spectra for f-TiO_2_ and F70-TiO_2_ (1:15): (A) Ti 2p, (B) O 1s and (C) C 1s; Figure S6: the GC-MS spectrogram for the oxidative coupling of benzylamine over F70-TiO_2_(1:15); Figure S7: The XRD signal peaks of F70-TiO_2_(1:15) recycled.

## Sample Availability

Samples of the compounds are not available from the authors.
