# Supplementary material for: Enhanced Photocatalytic Coupling of Benzylamine to N-Benzylidene Benzylamine over the Organic–Inorganic Composites F70-TiO2 Based on Fullerenes Derivatives and TiO2
Source: Molecules. 2023 May 24;28(11):4301. doi: 10.3390/molecules28114301 (PMC10254878; doi:10.3390/molecules28114301)
Supplement: Supplementary file 1 [file molecules-28-04301-s001.zip › molecules-2400980-supplementary.pdf]

Supplementary Information

Enhanced Photocatalytic Coupling of Benzylamine to N-Benzylidene Benzylamine over the Organic-Inorganic Composites F70-TiO_2 Based on Fullerenes Derivatives and TiO_2

Yanmeng Guo[1], Hang Li[1], Derui Kong, Bo Li[1], Xin Zhong[1], Yi-Fan Chen[1,*], Yujie Song[1,*]

[1]Hainan Provincial Key Laboratory of Fine Chemicals, College of Chemical Engineering and Technology, Hainan University, Haikou, China

*** Correspondence:**
Yi-Fan Chen
chenyifan@hainanu.edu.cn

Yujie Song
songyuejie@hainanu.edu.cn

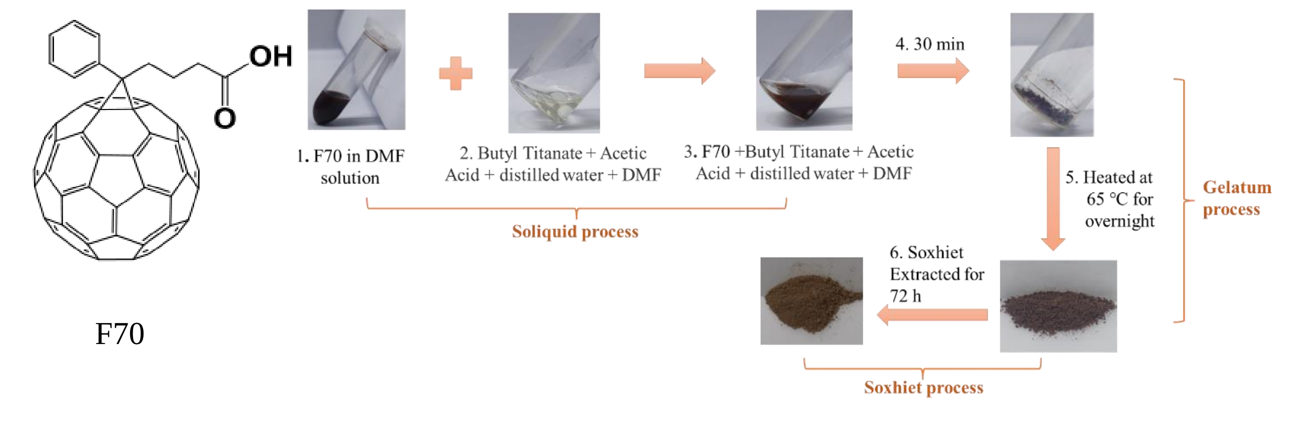

Figure S1. The structure of F70 and Synthesis of F70-TiO_2(1:15).

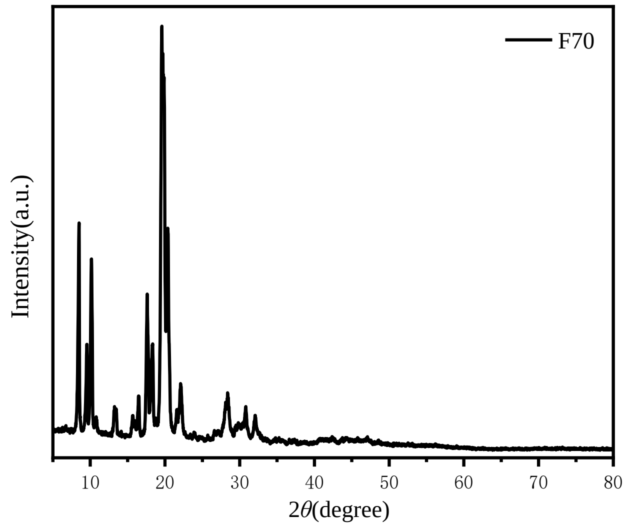

Figure S2. The XRD signal peaks of F70

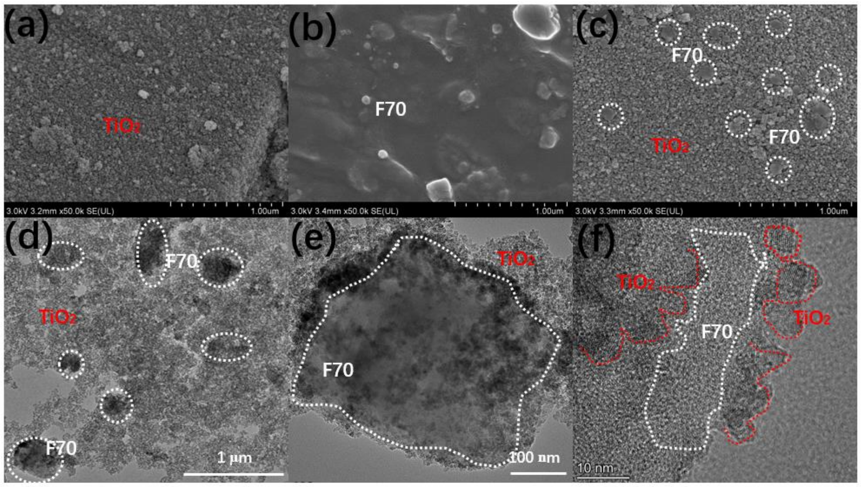

Figure S3. (a) SEM of *f*-TiO_2, (b) SEM of F70, (c) SEM, (d) TEM, and (e) HRTEM of F70-TiO_2(1:15).

Table S1. The BET surface area and the pore size distributions of *f*-TiO_2 and F70-TiO_2(1:15).

Sample	BET Surface area (m^2/g)	Pore size (nm)	Pore volume (cm^3/(g)
F70-TiO_2(1:15)	178.8	4.452	0.2380
f-TiO_2	198.6	4.386	0.2636

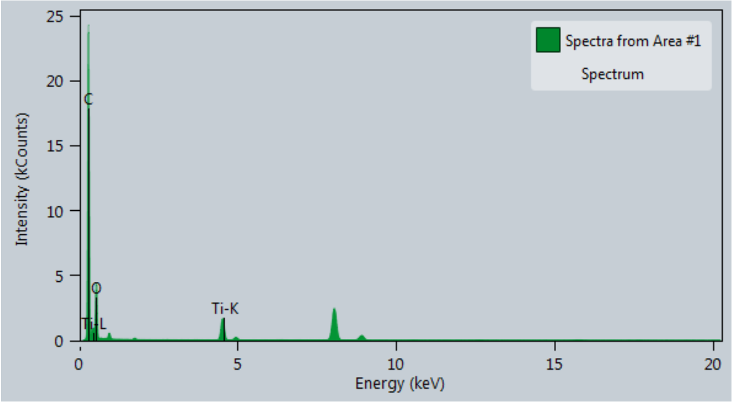

Figure S4. The EDX of F70-TiO_2(1:15).

Table S2. The Atom percentages and the binding energy of *f*-TiO_2 and F70-TiO_2(1:15) by the XPS analysis.

Sample (eV)	$Ti2p_{3/2}$ (eV)	$Ti2p_{1/2}$ (eV)	C1s	O1s
f-TiO_2	458.6	464.3	284.8<br>285.8<br>288.7	529.9<br>531.2
Atomic %	24.5		23.7	51.8
F70-TiO_2(1:15)	458.7	464.5	284.8<br>285.6<br>288.9	530.0<br>531.3
Atomic %	21.7		33.4	44.9

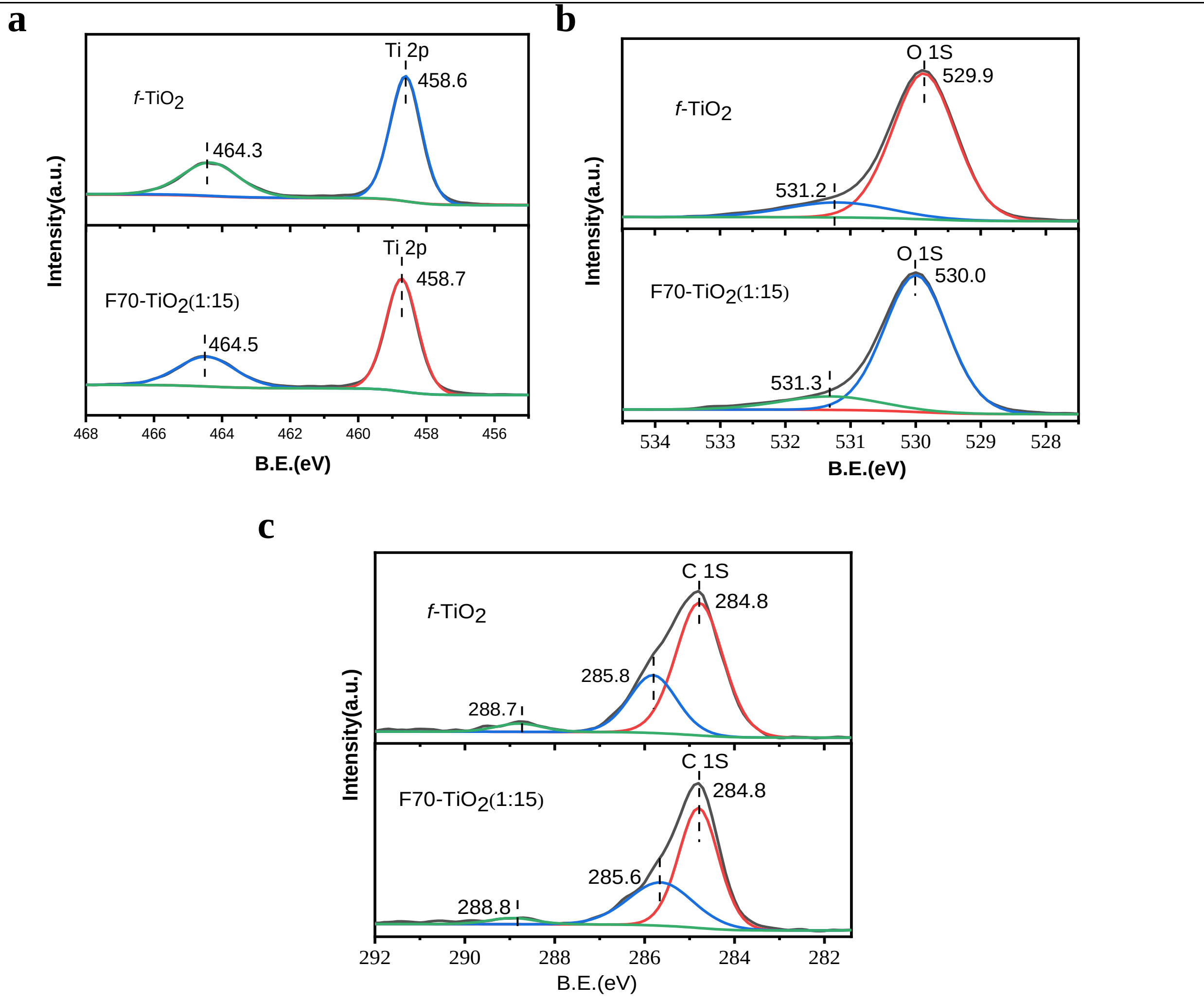

Figure S5. XPS spectra for *f*-TiO_2 and F70-TiO_2 (1:15): **(a)** Ti 2p, **(b)** O 1s and **(c)** C 1s.

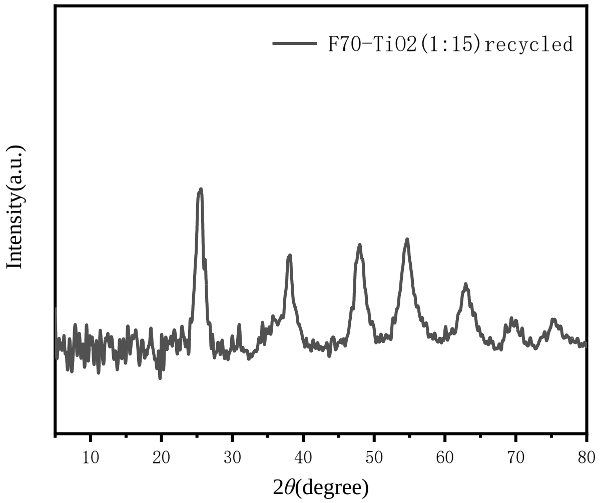

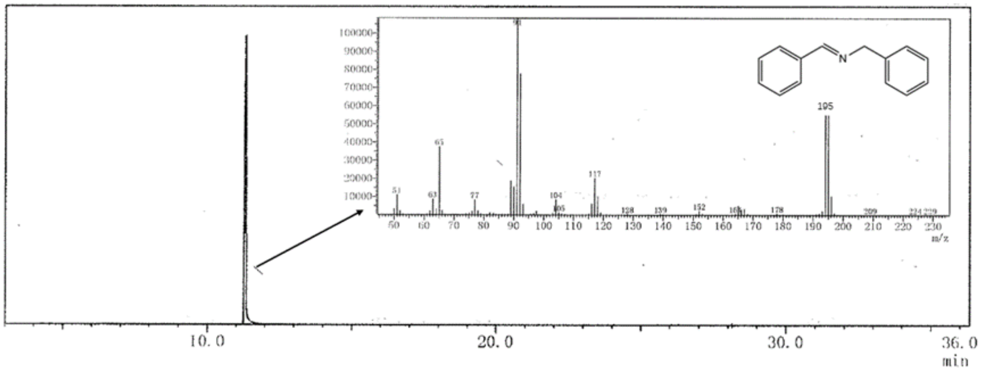

Figure S6. The GC-MS spectrogram for the oxidative coupling of benzylamine over F70-TiO_2(1:15).

Figure S7. The XRD signal peaks of F70-TiO_2(1:15) recycled.
